# Pharmacokinetics and Anti-Gastric Ulceration Activity of Oral Administration of Aceclofenac and Esomeprazole in Rats

**DOI:** 10.3390/pharmaceutics10030152

**Published:** 2018-09-06

**Authors:** Tae Hwan Kim, Subindra Kazi Thapa, Da Young Lee, Seung Eun Chung, Jun Young Lim, Hyeon Myeong Jeong, Chang Ho Song, Youn-Woong Choi, Sang-Min Cho, Kyu-Yeol Nam, Won-Ho Kang, Soyoung Shin, Beom Soo Shin

**Affiliations:** 1 College of Pharmacy, Catholic University of Daegu, Gyeongsan, Gyeongbuk 38430, Korea; thkim@cu.ac.kr; 2 College of Pharmacy, Wonkwang University, Iksan, Jeonbuk 54538, Korea; thapasubindra@gmail.com (S.K.T.); shins@wku.ac.kr (S.S.); 3 School of Pharmacy, Sungkyunkwan University, Suwon, Gyeonggi 16419, Korea; dayoung717@skku.edu (D.Y.L.); jsehome08@skku.edu (S.E.C.); panacea89@skku.edu (J.Y.L.); wise219143@skku.edu (H.M.J.); sky84312@skku.edu (C.H.S.); 4 Korea United Pharm. Inc., Seoul 06116, Korea; choi0528@kup.co.kr (Y.-W.C.); sweety1723@kup.co.kr (S.-M.C.); kynam@kup.co.kr (K.-Y.N.); kangwonho@kup.co.kr (W.-H.K.)

**Keywords:** aceclofenac, diclofenac, esomeprazole, pharmacokinetics, gastric ulcer

## Abstract

This study examined the effects of esomeprazole on aceclofenac pharmacokinetics and gastrointestinal complications in rats. Aceclofenac alone, or in combination with esomeprazole, was orally administered to male Sprague-Dawley rats. Plasma concentrations of aceclofenac, its major metabolite diclofenac, and esomeprazole were simultaneously determined by a novel liquid chromatography-tandem mass spectrometry method. Gastrointestinal damage was determined by measuring ulcer area and ulcer lesion index of the stomach. Oral administration of aceclofenac induced significant gastric ulceration, which was inhibited by esomeprazole administration. Following concurrent administration of aceclofenac and esomeprazole, overall pharmacokinetic profiles of aceclofenac and metabolic conversion to diclofenac were unaffected by esomeprazole. Aceclofenac metabolism and pharmacokinetics were not subject to significant food effects, whereas bioavailability of esomeprazole decreased in fed compared to fasting conditions. In contrast, the pharmacokinetics of aceclofenac and esomeprazole were significantly altered by different dosing vehicles. These results suggest that co-administration of esomeprazole with aceclofenac may reduce aceclofenac-induced gastrointestinal complications without significant pharmacokinetic interactions. The optimal combination and clinical significance of the benefits of the combination of aceclofenac and esomeprazole need to be further evaluated.

## 1. Introduction

Aceclofenac is one of the most popular oral nonsteroidal anti-inflammatory drugs (NSAIDs), and has proven effective in the treatment of osteoarthritis [1], rheumatoid arthritis [2], ankylosing spondylitis [3], and pain [4,5]. The absolute oral bioavailability of aceclofenac is 15% in rats [6] due to extensive metabolism. However, metabolism of aceclofenac is species-specific [7]. While aceclofenac is mainly metabolized to 4′-hydroxyaceclofenac in humans, the major metabolite in rats is diclofenac [7]. Aceclofenac and its metabolites exert anti-inflammatory effects by inhibition of prostaglandin E2 (PGE2) production via cyclooxygenases COX-1 and COX-2. Aceclofenac and 4′-hydroxyaceclofenac are selective COX-2 inhibitors with IC50 of 0.77 and 36 μM, respectively. Aceclofenac and 4′-hydroxyaceclofenac have IC50 > 100 μM for COX-1 in human whole blood [8]. In contrast, diclofenac inhibits both COX-1 (IC50 = 0.6 μM) and COX-2 (IC50 = 0.04 μM) [8]. Therefore, although diclofenac is present at lower levels in the plasma following aceclofenac administration in humans, diclofenac may contribute to the pharmacological action of aceclofenac in both humans and rats based on low IC50 values.

In general, selective COX-2 inhibitors exert less adverse effects on the gastrointestinal (GI) tract because COX-1 plays a protective role by stimulating the secretion of mucus and bicarbonate, increasing mucosal blood flow, and promoting epithelial proliferation [9]. Nevertheless, the superior clinical safety of aceclofenac related to GI adverse events is controversial. Aceclofenac was well tolerated, and the incidence of adverse GI events was generally lower than that of other NSAIDs [10]. Several clinical studies have shown that the overall withdrawal rate in aceclofenac recipients was lower than that in patients treated with tenoxicam [11], ketoprofen [2], and diclofenac [12]. Conversely, adverse events associated with aceclofenac use are still observed and include nausea, diarrhea, flatulence, gastritis, constipation, vomiting, and ulcerative stomatitis. No significant differences in GI event rates were reported between aceclofenac and other NSAIDs in individual clinical trials [1,2,3,11,12,13,14,15,16,17,18,19]. The number of patients with fecal blood loss did not differ significantly between aceclofenac and similar drugs such as tenoxicam [11,19], diclofenac [12,13], piroxicam [16], or indomethacin [3].

Gastroprotective therapy, such as with proton pump inhibitors (PPIs) in combination with NSAIDs, has been recommended for patients who are at risk for GI complications [20]. Among PPIs, esomeprazole, the S-isomer of omeprazole, has been shown to be useful in the prevention and healing of NSAID-induced gastric injury in clinical studies [21,22,23,24,25]. Nevertheless, co-therapy with NSAIDs and gastroprotective agents often results in poor clinical outcomes due to inadequate dosing and low patient compliance [26,27]. To improve patient compliance and clinical outcomes, a fixed-dose combination of an NSAID (naproxen) and esomeprazole magnesium (VIMOVO^®^) was approved by the United States FDA in 2010. 

Despite relatively low frequency and severity of GI complications with aceclofenac treatment alone, aceclofenac therapy may benefit when combined with esomeprazole. However, potential pharmacokinetic and pharmacodynamic interactions between aceclofenac and esomeprazole are not well characterized. To our knowledge, no reports are available on the effects of esomeprazole on the prevention or healing of aceclofenac-induced GI ulceration in preclinical and clinical studies. 

Therefore, the present study evaluated pharmacokinetic interactions between aceclofenac and esomeprazole, as well as effects of esomeprazole on aceclofenac-induced gastric ulceration in rats. Food effects on the pharmacokinetics of aceclofenac and esomeprazole were also studied.

## 2. Materials and Methods 

### 2.1. Chemicals

Aceclofenac and esomeprazole magnesium dihydrate were supplied by Korea United Pharm. Inc. (Seoul, Korea). Diclofenac, lansoprazole, ammonium formate, sodium bicarbonate, and methylcellulose were purchased from Sigma-Aldrich Co. (St. Louis, MO, USA). HPLC grade acetonitrile, methanol, and distilled water were purchased from J.T. Baker, Inc. (Philipsburg, NJ, USA). Polyethylene glycol (PEG) 200 and citric acid were obtained from Junsei Chemical Co. (Tokyo, Japan), dimethyl sulfoxide (DMSO) was purchased from Kanto Chemical Co. (Tokyo, Japan), and urea was purchased from USB Co. (Cleveland, OH, USA). 

### 2.2. Animals

Male Sprague-Dawley rats (8–10 weeks; body weight 220–282 g) were purchased from Samtako (Osan, Korea) and kept in plastic cages with free access to water and standard rat diet. The rats were maintained at a temperature of 23 ± 2 °C with a 12 h light-dark cycle and relative humidity of 50 ± 10%. They were acclimatized for at least one week prior to experimentation. All animal care and the protocols were conducted according to the Guidelines for the Care and Use of Animals which was approved by the Catholic University of Daegu (IACUC-2016-006; 29 April 2016) and Wonkwang University (WKU17-02; 10 January 2017). 

### 2.3. Pharmacokinetic Studies

Aceclofenac and esomeprazole were prepared in four different vehicles, i.e., DMSO:PEG200:distilled water = 10:70:20 (vehicle A), 0.5% methylcellulose suspension in distilled water (vehicle B), citric urea buffer which consisted of urea (20 g/mL) and trisodium citrate (10 g/mL) for aceclofenac and 0.5% methylcellulose suspension containing 0.25 mM NaHCO_3_ for esomeprazole (vehicle C), and DMSO:citric urea buffer = 1:14 solution for aceclofenac and DMSO:citric urea buffer = 1:9 solution for esomeprazole (vehicle D). 

The rats were fasted for 12 h prior to the drug administration except for the rats for the fed condition in which the rats were allowed free access to standard rat diet to examine food effect. Aceclofenac and esomeprazole solutions or suspensions were administered to rats by oral gavage. For vehicles A and B, aceclofenac and esomeprazole were dissolved or suspended together in the vehicle and simultaneously administered to rats. For vehicle C, aceclofenac in the citric urea buffer, esomeprazole in 0.5% methylcellulose suspension, and 0.25 mM NaHCO_3_ were sequentially administered with a 5 min interval. For vehicle D, esomeprazole solution was administered first, and aceclofenac solution was administered 10 min after the esomeprazole dose. Approximately 200 μL of venous blood was collected predose and 0.25, 0.5, 1, 2, 3, 4, 6, 8, 12, 18, and 24 h postdose from the jugular vein. Plasma samples were obtained by centrifugation of the blood samples at 4000× *g* for 10 min and stored at −70 °C until analysis. The stomach was collected for measurement of gastric lesions.

### 2.4. Measurement of Gastric Lesions

To evaluate the effects of esomeprazole on gastric damage associated with aceclofenac administration, rats were separated into six groups, i.e., control group (blank vehicle D, *n* = 7), positive control group (200 mg/kg of aceclofenac, *n* = 7), and four test groups (200 mg/kg of aceclofenac + 5, 10, 20, or 40 mg/kg of esomeprazole, *n* = 3 or 4). The rats were fasted 24 h before aceclofenac and esomeprazole doses. Esomeprazole solution was administered 10 min prior to aceclofenac (200 mg/kg) administration. At 6 h after the aceclofenac dose, rats were sacrificed, the stomach was excised, and gastric damage was examined. Since this study was conducted by two separate experiments and each experiment has the control and positive control, the number of animals included for the control (*n* = 7) and positive control (*n* = 7) was higher than test groups (*n* = 3–4). Except for the esomeprazole doses, all experimental protocols were identical between the two studies.

The gastric damage by aceclofenac was measured by calculation of ulcerative lesion area and ulcer index. After sacrificing the animal, the stomach was dissected along its greater curvature and fixed on a board. The stomach was then macroscopically examined, and the area of the lesion was quantified using an image analysis program, Image J (National Institutes of Health, Bethesda, MD, USA). The extent of ulceration was also assessed by calculation of an ulcer index according to the method of Andrade et al. [28,29]. Ulcers were first classified as ulcer area < 1 mm^2^ (Level I), ulcer area = 1–3 mm^2^ (Level II), and ulcer area > 3 mm^2^ (Level III), and the ulcer indexes were calculated:

$$\begin{array}{l} \mathrm{Ulcer}\;\mathrm{index}\\ =1\times (\mathrm{number}\;\mathrm{of}\;\mathrm{Level}\;I\;\mathrm{ulcer})+2\times (\mathrm{number}\;\mathrm{of}\;\mathrm{Level}\;\mathrm{II}\;\mathrm{ulcer})\;+\;3\times (\mathrm{number}\;\mathrm{of}\;\mathrm{Level}\;\mathrm{III}\;\mathrm{ulcer}) \end{array}$$

### 2.5. Liquid Chromatography-Tandem Mass Spectrometry (LC-MS/MS)

Concentrations of aceclofenac, its metabolite, diclofenac, and esomeprazole in the rat plasma were determined by a newly developed LC-MS/MS assay. Calibration samples were prepared by spiking 50 μL of blank rat plasma with 50 μL of the standard aceclofenac, diclofenac, and esomeprazole working standard solutions in methanol followed by addition of 50 μL of the internal standard (IS) solution (lansoprazole, 100 ng/mL in methanol) and 350 μL of methanol as precipitation solvent. The final calibration samples yielded concentrations of 5, 10, 50, 100, 500, 1000, 5000, and 10,000 ng/mL for aceclofenac, 10, 50, 100, 500, 1000, 5000, and 10,000 ng/mL for diclofenac, and 0.25, 0.5, 2.5, 5, 25, 50, 250, and 500 ng/mL for esomeprazole. For plasma sample preparation, 50 μL of the IS and 400 μL of methanol were added to 50 μL of the rat plasma samples. The mixture was then mixed on a vortex mixer for 1 min and centrifuged at 34,220× *g* for 10 min. After centrifugation, 100 μL of the supernatant was diluted with the same volume of 25 mM ammonium formate, and a volume of 2 μL was injected into the LC-MS/MS. 

The LC-MS/MS comprised an Agilent 6430 coupled with an Agilent 1200 HPLC system (Agilent, Santa Clara, CA, USA). Chromatographic separations were achieved on a Kinetex C18 column 50 × 2.10 mm i.d., 2.0 μm (Phenomenex, Torrence, CA, USA) with a Security Guard Cartridge (Phenomenex, Torrence, CA, USA). The mobile phase (MP) consisted of a mixture of 25 mM ammonium formate with 0.2% formic acid (MP-A) and acetonitrile (MP-B). The initial condition of 77% MP-A and 23% MP-B was held for 4 min followed by a linear increase of MP-B to 90% in the next 0.3 min. The flow rate of the mobile phase was set at 0.35 mL/min at the initial condition. The composition of 10:90 of MP-A:MP-B was maintained for 1 min, and the flow rate was 0.6 mL/min. The system was brought back to the initial condition of 77% MP-A by a linear gradient in 0.3 min and a flow rate of 0.35 mL/min and maintained for 3 min before the next injection. The column oven temperature was 40 °C. The mass spectrometer was operated using electron spray ionization (ESI) in positive ion mode with a dwell time of 100 ms. The fragmentor voltage was 85 V for aceclofenac, esomeprazole, and the IS, and 80 V for diclofenac. The collision energy was 40 V for aceclofenac, 34 V for diclofenac, 5 V for esomeprazole, and 5 V for the IS. Gas temperature, gas flow rate, and nebulizer gas pressure were set at 350 °C, 10 L/min, and 20 psi, respectively. The transition of the precursors to the product ion was monitored at *m/z* 354.0→214.0 for aceclofenac, 296.0→214.0 for diclofenac, 346.3→198.0 for esomeprazole, and 370.1→252.0 for the IS. 

The lower limit of quantification (LLOQ) of the assay was 5 ng/mL, 10 ng/mL, and 0.25 ng/mL for aceclofenac, diclofenac, and esomeprazole, respectively, in the rat plasma. The intra- and inter-day accuracy was 103.0–110.8% for aceclofenac, 95.2–108.8% for diclofenac, and 90.4–111.0% for esomeprazole (*n* = 4, each). The intra- and inter-day precision was below 11.0% for aceclofenac, 12.1% for diclofenac, and 13.2% for esomeprazole (*n* = 4, each). The recovery calculated by comparing the peak area obtained from the standard solution spiked in the blank plasma followed by a protein precipitation process to that obtained from the matrix-free solvent was 97.2 ± 6.8% for aceclofenac, 89.9 ± 5.0% for diclofenac, 109.3 ± 2.0% for esomeprazole, and 107.5 ± 3.1% for the IS (*n* = 3). 

### 2.6. Non-Compartmental Analysis

The non-compartmental pharmacokinetic parameters were estimated by Phoenix^®^ WinNonlin^®^ 6.4 (Certara, Princeton, NJ, USA). These parameters included terminal half-life (*t*_1/2_), peak plasma concentration (*C*_max_) and time to reach *C*_max_ (*T*_max_), areas under the plasma concentration vs. time curve from time zero to the last observation time (AUC_all_) and from time zero to infinity (AUC_∞_), and systemic clearance (CL/F). 

### 2.7. Statistical Analysis

The obtained data were analyzed with unpaired *t*-tests for comparisons between two means of unpaired data or one-way analyses of variance followed by Tukey’s post hoc test for comparisons among more than two means of unpaired data. Data were expressed as the mean ± standard deviation (SD). The statistical significance level was set at *p* < 0.05 (SPSS Statistics 17.0, SPSS Inc., Chicago, IL, USA).

## 3. Results

### 3.1. Effects of Esomeprazole on Aceclofenac Induced Gastric Damage

**Figure 1:**
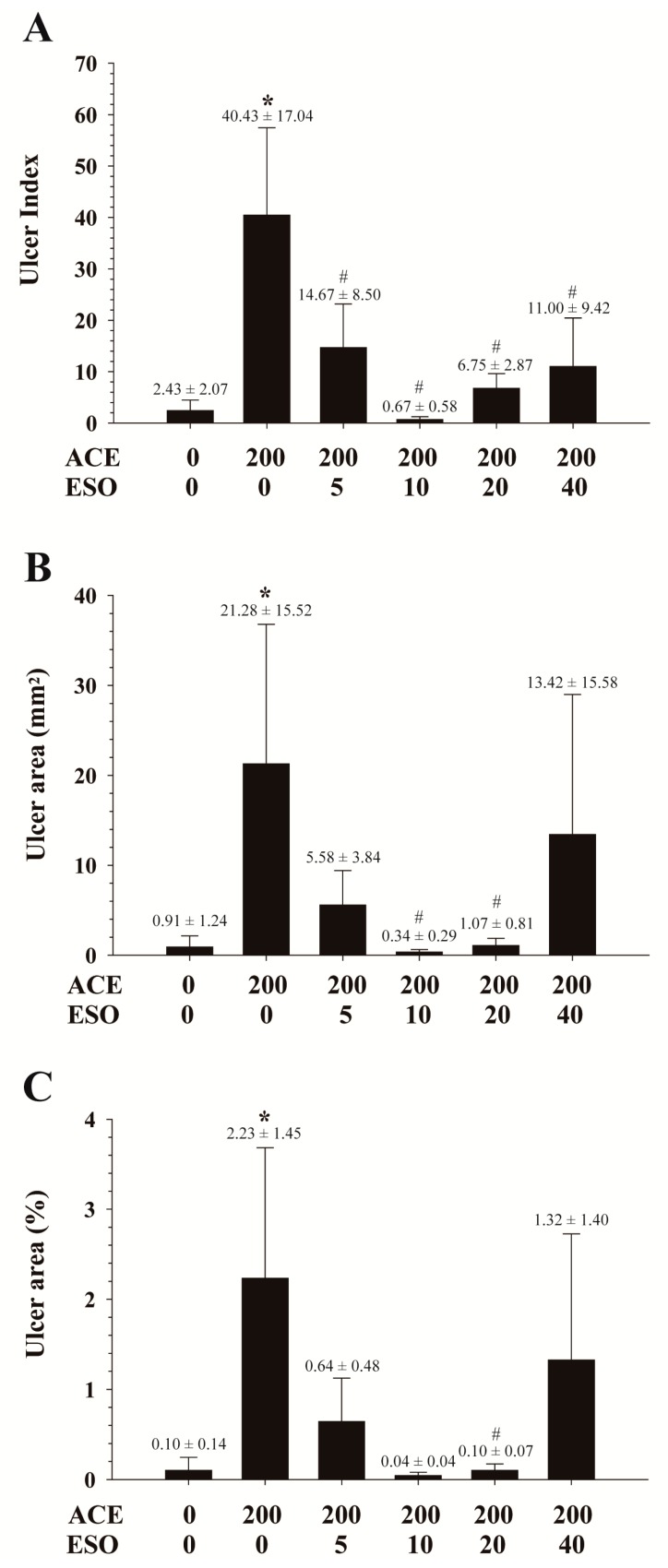
Aceclofenac (ACE) 200 mg/kg-induced gastric ulcers represented by (**A**) ulcer index, (**B**) ulcer area (mm^2^), and (**C**) relative ulcer area (%) in the absence or in the presence of pre-treatments with esomeprazole (ESO) 0, 5, 10, 20, 40 mg/kg in rats. Each column represents the mean ± SD. *, *p* < 0.05 vs. control (ACE = 0/ESO = 0 mg/kg); ^#^, *p* < 0.05 vs. ACE = 200/ESO = 0 mg/kg.

Figure 1 shows ulcer index and ulcer areas following oral administration of aceclofenac (200 mg/kg) with or without esomeprazole pre-treatments (0, 5, 10, 20, 40 mg/kg). Two separate experiments were conducted, and data from the two studies were pooled together. While minimal gastric ulcers were observed after administration of control vehicle, significantly higher ulcer index and ulcer area were observed following aceclofenac administration at 200 mg/kg. Ulcer index was significantly reduced by administration of esomeprazole (Figure 1A). However, no statistically significant dose-dependent differences across esomeprazole doses of 5–40 mg/kg were observed, and increasing the dose of esomeprazole to 40 mg/kg did not result in an increased reduction of aceclofenac-induced gastric lesions. Both absolute gastric ulcer area (mm^2^) and relative ulceration area compared to total stomach area (%) showed a trend similar to the ulcer index. Significantly higher ulcer areas were observed with aceclofenac treatment, with reduction by esomeprazole co-administration at 10 and 20 mg/kg (Figure 1B,C).

### 3.2. Effects of Esomeprazole on Aceclofenac Pharmacokinetics

**Figure 2:**
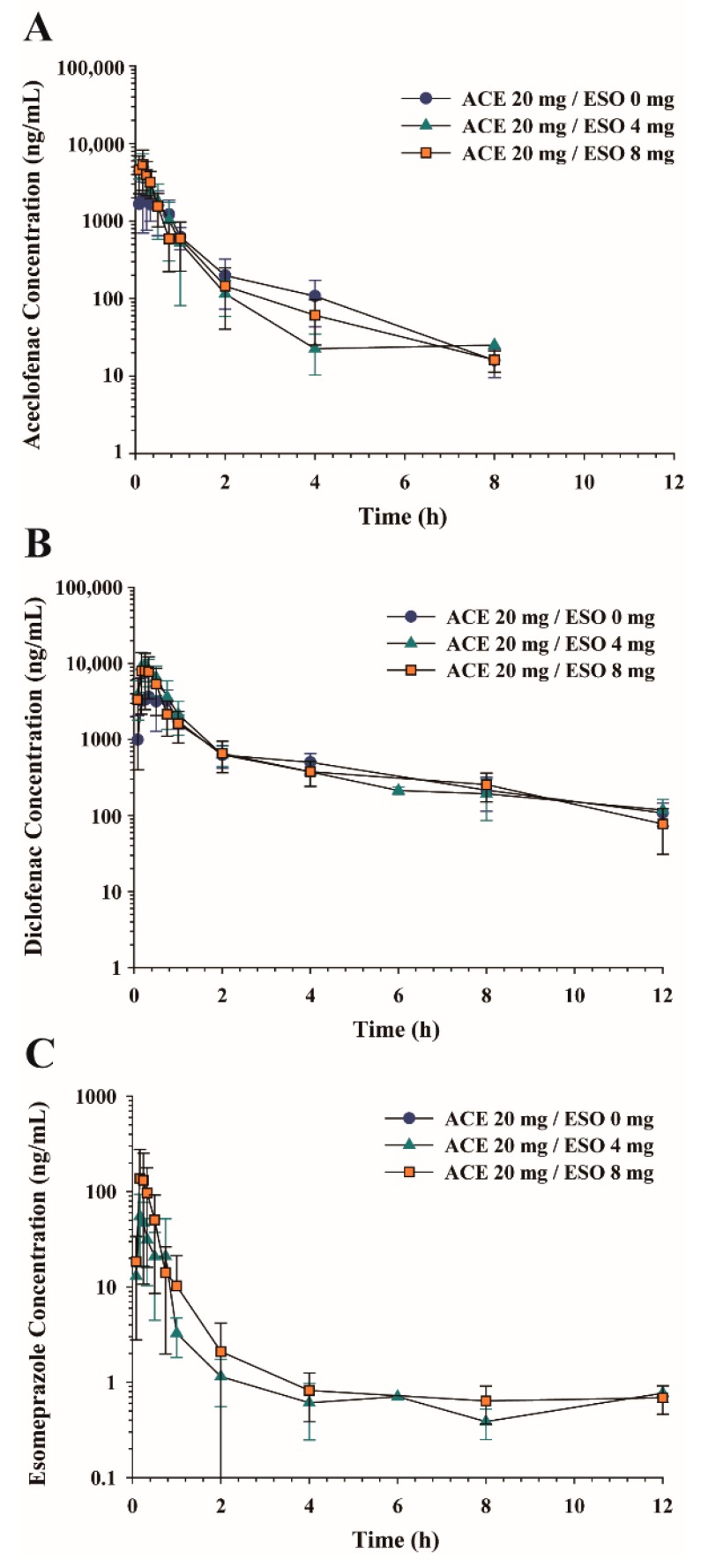
Average plasma concentration-time profiles of (**A**) aceclofenac, (**B**) diclofenac, and (**C**) esomeprazole following oral administration of aceclofenac (20 mg/kg) in combination with esomeprazole (0, 4, 8 mg/kg) in rats. Data represent mean ± SD (*n* = 4–6).

Effects of esomeprazole on aceclofenac pharmacokinetics were evaluated after oral administration of aceclofenac (20 mg/kg) in combination with esomeprazole (0, 4, 8 mg/kg) delivered with vehicle C in rats. Average plasma concentration vs. time profiles of aceclofenac, diclofenac, and esomeprazole are shown in Figure 2, and non-compartmental pharmacokinetic parameters are summarized in Table 1. Plasma aceclofenac concentration rapidly increased and reached *C*_max_ within 0.23 h. Plasma concentration then declined with a mean terminal half-life (*t*_1/2_) of 0.94–1.39 h. No significant differences in aceclofenac pharmacokinetic parameters were found with esomeprazole co-administration (Table 1). 

Diclofenac, a metabolite of aceclofenac, plasma concentrations also rapidly increased following aceclofenac administration to maximum concentrations (*C*_max_) of 4.91–10.76 μg/mL with mean *T*_max_ of 0.23–0.50 h. *C*_max_ as well as AUC of diclofenac were higher than those of aceclofenac. The *t*_1/2_ of diclofenac was 3.24–3.84 h. Pharmacokinetic parameters of diclofenac were unaltered by esomeprazole co-administration. The fraction of metabolite formation (AUC_DIC_/AUC_ACE_) was also unaltered by esomeprazole administration.

Following oral co-administration of aceclofenac and esomeprazole, esomeprazole plasma concentration rapidly increased and reached *C*_max_ within 0.15 h, then declined with a *t*_1/2_ of 3.87–5.67 h (Figure 2C and Table 1). While the *t*_1/2_ and *T*_max_ were similar following 4 and 8 mg/kg esomeprazole administration, average *C*_max_, AUC_all_, and AUC_∞_ proportionally increased with the dose increase.

### 3.3. Effects of Different Dosing Vehicles on the Pharmacokinetics of Aceclofenac and Esomeprazole

**Figure 3:**
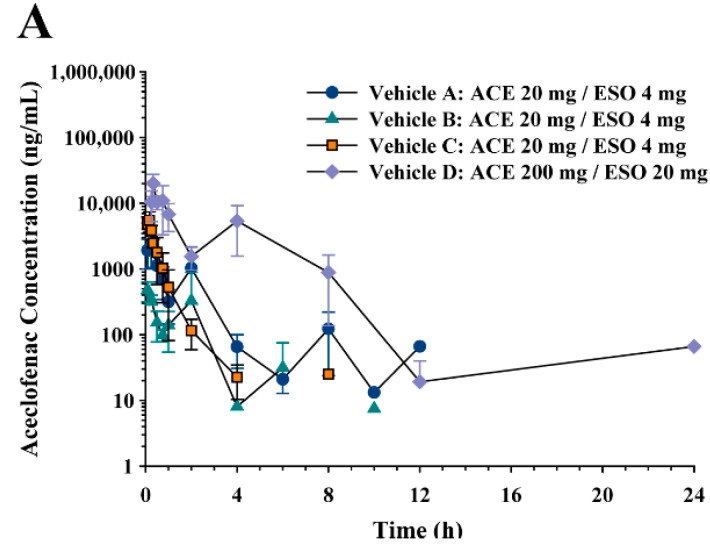

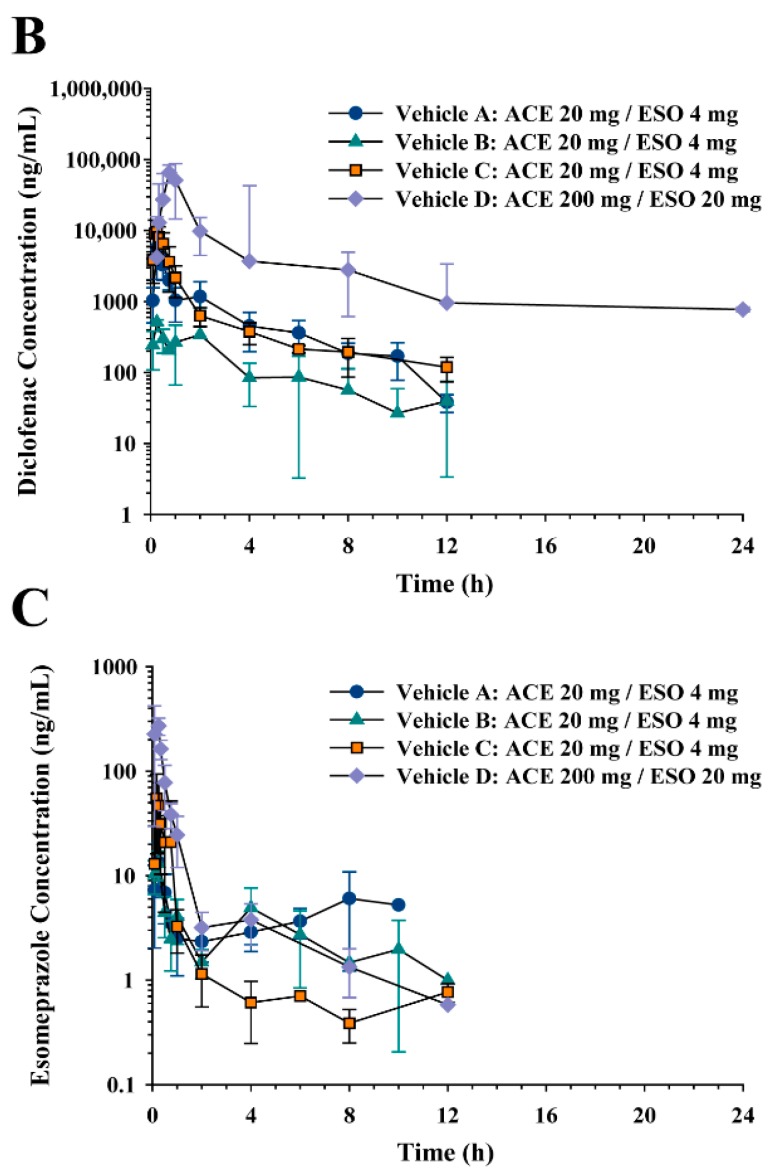
Average plasma concentration-time profiles of (**A**) aceclofenac, (**B**) diclofenac, and (**C**) esomeprazole following oral administration of vehicle A, B, C, and D containing aceclofenac (20 mg/kg for A, B, and C and 200 mg/kg for D) and esomeprazole (4 mg/kg for A, B, and C and 20 mg/kg for D) in rats.

Figure 3 summarizes plasma concentration vs. time profiles of aceclofenac, diclofenac, and esomeprazole following administration of aceclofenac and esomeprazole using different dosing vehicles. The non-compartmental pharmacokinetic parameters are summarized in Table 2. Since different doses were used with vehicles A, B, C (aceclofenac 20 mg/kg and esomeprazole 4 mg/kg), and vehicle D (aceclofenac 200 mg/kg and esomeprazole 20 mg/kg), the systemic exposure parameters *C*_max_ and AUC were normalized by dose and compared. Statistical comparisons were made only for dose-independent parameters, such as *t*_1/2_, *T*_max_, *C*_max_/D, AUC/D, and CL/F.

For aceclofenac, significant differences were found for *t*_1/2_, *C*_max_/D, and AUC_all_/D depending on the vehicle. Drug *t*_1/2_ was longer when vehicle B (0.5% methylcellulose suspension in distilled water) was used than when vehicle C was used. Aceclofenac *C*_max_/D as well as AUC_all_/D with vehicle B was also lower than those in other vehicles. The *C*_max_/D of aceclofenac was the highest in vehicle C compared to all other vehicles. However, the AUC_∞_/D of aceclofenac was comparable across all tested vehicles.

Most pharmacokinetic parameters of diclofenac except *t*_1/2_ were similar regardless of the dosing vehicle. The fraction metabolized, i.e., AUC_DIC_/AUC_ACE_, after aceclofenac administration was higher in vehicle C compared to that in vehicle B. 

Most pharmacokinetic parameters of esomeprazole were not changed by different dosing vehicles, except *C*_max_/D. The *C*_max_/D values were similar between vehicles C and D, which were over 3.7-fold higher than those in vehicles A and B (Table 2). 

### 3.4. Food Effects on the Pharmacokinetics of Aceclofenac and Esomeprazole

**Figure 4:**
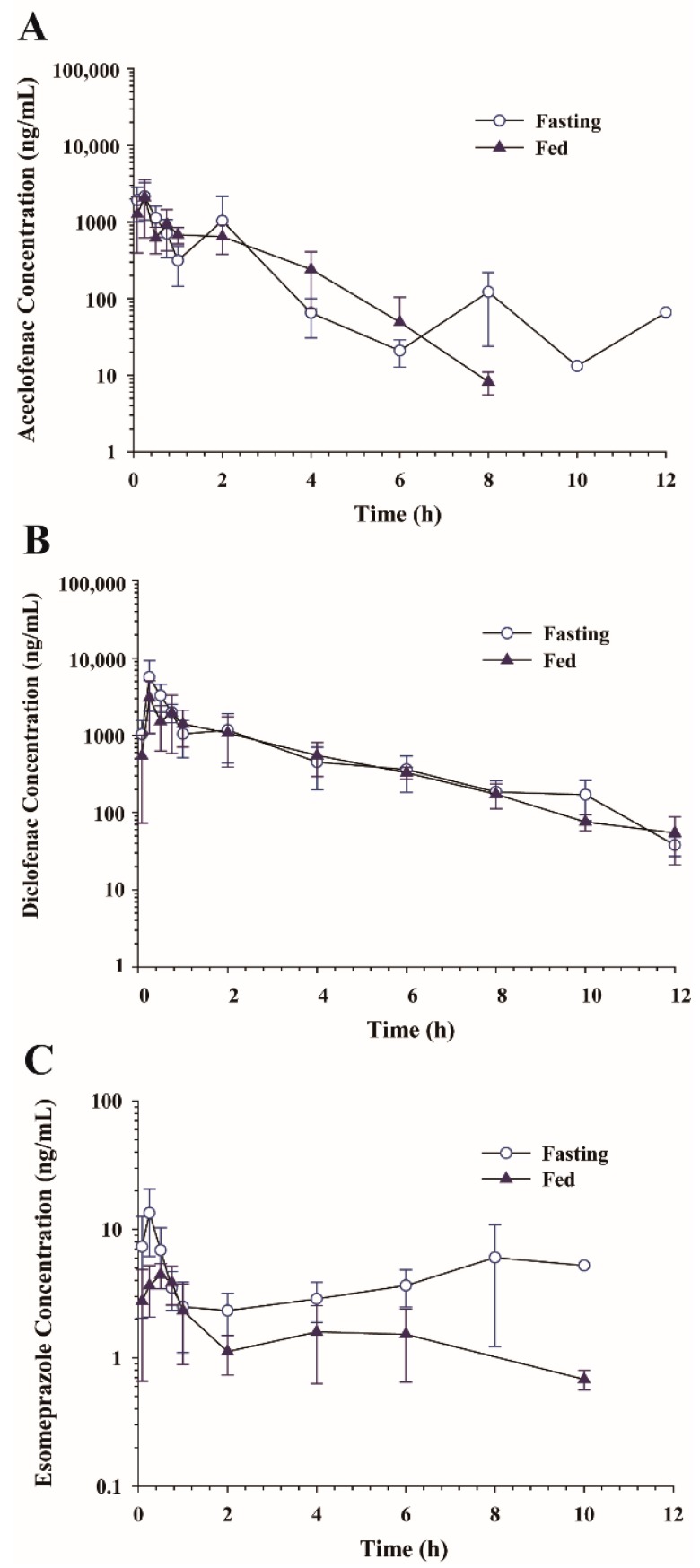
Average plasma concentration-time profiles of (**A**) aceclofenac, (**B**) diclofenac, and (**C**) esomeprazole following oral administration of aceclofenac (20 mg/kg) in combination with esomeprazole (4 mg/kg) in rats with fasting vs. fed conditions. Data represent mean ± SD (*n* = 4–5).

To evaluate food effects on aceclofenac and esomeprazole pharmacokinetics, aceclofenac (20 mg/kg) and esomeprazole (4 mg/kg) prepared in vehicle A were orally administered in fasting and fed conditions. Plasma concentration vs. time profiles of aceclofenac, diclofenac, and esomeprazole are shown in Figure 4. Non-compartmental pharmacokinetic parameters of aceclofenac, diclofenac, and esomeprazole in fasting and fed conditions are summarized in Table 3. Although overall plasma concentration vs. time profiles of aceclofenac and diclofenac were similar between fasting and fed conditions (Figure 4A,B), a longer t_1/2_ was observed in fasting conditions for aceclofenac. In contrast, there was no significant difference between the *t*_1/2_ in the fasting and fed conditions for diclofenac (Table 3). Conversely, overall esomeprazole plasma concentrations were lower in the fed condition than in the fasting condition (Figure 4C). The *t*_1/2_ was longer and *C*_max_ and AUC_all_ were significantly lower for esomeprazole in the fed condition (Table 3).

## 4. Discussion

This study demonstrated no significant pharmacokinetic interactions between aceclofenac and esomeprazole in rats. Metabolic formation of diclofenac from aceclofenac was fast and extensive, which was consistent with previous reports [6,7]. Aceclofenac metabolism to diclofenac was also largely unaffected by concurrent administration of esomeprazole. Although diclofenac may be present at lower levels in humans than in rats, it significantly inhibits enzymatic activity of COX enzymes and contributes to the pharmacological action of aceclofenac in both humans and rats. Moreover, consistent with results in humans [22,23,25], our results indicate that esomeprazole may suppress gastric mucosal damage induced by aceclofenac in rats. 

Substantial lesions were observed in the gastric mucosa following single dose oral administration of aceclofenac at 200 mg/kg in rats compared to that following vehicle administration (Figure 1). Co-administration of esomeprazole significantly suppressed gastric mucosal damage. Similarly, esomeprazole has demonstrated efficacy in the prevention and treatment of NSAIDs-induced gastric ulcers in humans [22,23,25] and experimental animals [30]. However, esomeprazole prevention of gastric ulcers was not dose-dependent in our study (Figure 1). While gastric ulceration induced by aceclofenac was almost entirely prevented by co-administration with esomeprazole at 10 and 20 mg/kg, no additional benefit was seen at the higher esomeprazole dose (40 mg/kg).

The gastro-protective effects of esomeprazole in combination with NSAIDs are potentially attributed to multiple mechanisms. Both acid-dependent and acid-independent mechanisms were proposed. Esomeprazole significantly inhibited gastric acid secretion in parallel with a significant reduction in mucosal damage in rats treated with indomethacin for 2 weeks [30]. In addition, esomeprazole restored proliferating cell nuclear antigen (PCNA) and Ki-67 expression and decreased malondialdehyde (MDA) levels and caspase-3 expression [30]. Reduction of oxidative tissue damage may also contribute to the gastroprotective actions of esomeprazole [31,32]. Digestive effects of esomeprazole may also confer gastroprotection [31]. Although the present study mainly focused on apparent gastric injury, we also analyzed the biomarkers that may contribute to gastric injury in mucosal samples. However, measured levels of MDA and protein expression of NF-κB, PCNA, and caspase-3 were variable and not dependent on aceclofenac administration. Minor changes in these biomarkers were observed with esomeprazole co-administration. It is possible that mucosal samples may be damaged during macroscopic examination in the present experimental setting. In addition, experimental conditions (i.e., a single dose administration of aceclofenac and esomeprazole) are different from studies that examined the effects of repeated exposures of indomethacin for two weeks followed by one week of esomeprazole co-administration [30]. The mechanism of gastroprotective effects of esomeprazole in combination with aceclofenac should be evaluated in further studies. 

When pharmacokinetics of aceclofenac and esomeprazole were compared in fed vs. fasted conditions, the *t*_1/2_ of aceclofenac was longer in fasting conditions. Multiple peaks were observed in the plasma concentration vs. time profiles and were more prominent in the fasting conditions (Figure 4A). These results agree with those of previous studies in which multiple peaks were present [6]. The multiple-peak phenomenon may result from the presence of either enterohepatic recirculation or a complicated absorption process [33,34], which may be favorable in fasting conditions [6]. The pharmacokinetics of the metabolite diclofenac were not altered by the presence of food. However, a significant reduction in systemic exposure to esomeprazole was observed in the fed versus fasting condition (Table 3). This is consistent with previous reports that the mean exposure after administration of a single dose of esomeprazole was decreased by 43–53% after food intake compared to fasting conditions resulting in the recommendation for esomeprazole to be taken at least one hour before meals [35]. 

During the present study, we also found that both aceclofenac and esomeprazole pharmacokinetics were significantly dependent on dosing vehicles. Among four different dosing vehicles, vehicle C (citric urea buffer solution) allowed for the most rapid absorption of aceclofenac, as evidenced by the highest *C*_max_/D with a short *T*_max_ (Table 2). A previous study reported that a mixture of urea and sodium citrate similar to vehicle C markedly increased aceclofenac water solubility [6]. Increased water solubility may have contributed to the increased rate of aceclofenac absorption. On the contrary, vehicle B (0.5% methylcellulose suspension in distilled water) induced lower *C*_max_/D and AUC_all_/D with a longer *t*_1/2_ compared to the solution vehicle, which may be due to slower absorption of the suspension. However, the overall extent of absorption and AUC_∞_ of aceclofenac were comparable across suspension and solution vehicles. For esomeprazole, a significantly higher *C*_max_ was observed when esomeprazole was dosed in vehicles C and D than in vehicles A and B (Table 2). Esomeprazole stability is pH-dependent, and it degrades rapidly in an acidic environment [35]. Vehicles C and D contained neutral buffers, which may have prevented acid-catalyzed degradation of esomeprazole in the stomach leading to a higher *C*_max_. When omeprazole was given in combination with sodium bicarbonate, bioavailability was reported to increase by 2-fold [36].

## 5. Conclusions

Potential interactions between aceclofenac and esomeprazole were evaluated with regard to pharmacokinetics and GI adverse effects. While metabolism and pharmacokinetic profiles of aceclofenac were mostly unaltered, gastric ulceration associated with aceclofenac was significantly inhibited by concurrent administration of esomeprazole. The clinical benefits, as well as the optimal dose of the combination of aceclofenac and esomeprazole, should be evaluated in further studies. Since both aceclofenac and esomeprazole absorption was affected by dosing vehicles, design of optimal formulations may provide further advantages with regard to pharmacokinetics and efficacy.

## Figures and Tables

**Table 1:** Non-compartmental pharmacokinetic parameters of aceclofenac, diclofenac, and esomeprazole obtained after oral administration of aceclofenac (20 mg/kg) and esomeprazole (0, 4 and 8 mg/kg) to rats. Parameters include terminal half-life (*t*_1/2_), peak plasma concentration (*C*_max_), time to reach *C*_max_ (*T*_max_), areas under the plasma concentration vs. time curve from time zero to the last observation time (AUC_all_) and from time zero to infinity (AUC_∞_), and systemic clearance (CL/F).

Compound	Parameters	ACE 20/ESO 0 mg/kg (*n* = 4)	ACE 20/ESO 4 mg/kg (*n* = 6)	ACE 20/ESO 8 mg/kg (*n* = 4)
Aceclofenac	*t*_1/2_ (h)	1.38 ± 0.21	0.94 ± 0.70	1.39 ± 0.72
	*T*_max_ (h)	0.23 ± 0.20	0.17 ± 0.07	0.19 ± 0.10
	*C*_max_ (μg/mL)	3.07 ± 1.20	5.12 ± 1.66	5.72 ± 2.55
	AUC_all_ (μg·h/mL)	2.30 ± 0.54	2.54 ± 0.51	2.79 ± 0.61
	AUC_∞_ (μg·h/mL)	2.36 ± 0.52	2.57 ± 0.51	2.83 ± 0.60
	CL/F (mL/min/kg)	146.80 ± 32.40	134.77 ± 32.32	121.94 ± 26.32
Diclofenac	*t*_1/2_ (h)	3.84 ± 0.32	3.82 ± 1.65	3.24 ± 0.98
	*T*_max_ (h)	0.50 ± 0.30	0.28 ± 0.11	0.23 ± 0.08
	*C*_max_ (μg/mL)	4.91 ± 3.19	10.76 ± 2.74	8.89 ± 4.98
	AUC_all_ (μg·h/mL)	7.16 ± 1.98	9.44 ± 1.1	8.56 ± 3.32
	AUC_∞_ (μg·h/mL)	7.76 ± 2.13	10.1 ± 1.35	8.97 ± 3.24
	AUC_DIC_/AUC_ACE_	3.94 ± 1.73	4.64 ± 1.32	3.63 ± 0.97
Esomeprazole	*t*_1/2_ (h)	-	3.87 ± 2.21	5.67 ± 3.68
	*T*_max_ (h)	-	0.15 ± 0.14	0.13 ± 0.05
	*C*_max_ (μg/mL)	-	57.14 ± 25.94	142.75 ± 132.71
	AUC_all_ (μg·h/mL)	-	27.68 ± 13.39	64.02 ± 49.78
	AUC_∞_ (μg·h/mL)	-	30.91 ± 12.76	68.71 ± 48.72
	CL/F (mL/min/kg)	-	2442.37 ± 884.54	3087.76 ± 2334.25

**Table 2:** Non-compartmental pharmacokinetic parameters of aceclofenac and diclofenac obtained after oral administration of vehicle A, B, C, and D containing aceclofenac (20 mg/kg for A, B, and C and 200 mg/kg for D) and esomeprazole (4 mg/kg for A, B, and C and 20 mg/kg for D) to rats.

Compound	Parameters	Vehicle A (*n* = 5)	Vehicle B (*n* = 4)	Vehicle C (*n* = 6)	Vehicle D (*n* = 6)
Aceclofenac	*t*_1/2_ (h) ^a)^	2.13 ± 0.30	6.47 ± 5.59	0.94 ± 0.70	1.89 ± 1.65
	*T*_max_ (h)	0.57 ± 0.80	0.60 ± 0.93	0.17 ± 0.07	0.27 ± 0.21
	*C*_max_ (μg/mL)	2.53 ± 0.57	0.69 ± 0.33	5.12 ± 1.66	21.69 ± 6.48
	*C*_max_ (μg/mL)/D (mg/kg) ^b)^	0.13 ± 0.03	0.03 ± 0.02	0.26 ± 0.08	0.11 ± 0.03
	AUC_all_ (μg·h/mL)	3.35 ± 1.72	0.88 ± 0.82	2.54 ± 0.51	34.75 ± 9.82
	AUC_all_ (μg·h/mL)/D (mg/kg) ^c)^	0.37 ± 0.07	0.07 ± 0.06	0.47 ± 0.05	0.63 ± 0.23
	AUC_∞_ (μg·h/mL)	3.53 ± 1.89	1.42 ± 1.14	2.57 ± 0.51	38.16 ± 9.00
	AUC_∞_ (μg·h/mL)/D (mg/kg)	0.18 ± 0.09	0.07 ± 0.06	0.13 ± 0.03	0.19 ± 0.05
	CL/F (mL/min/kg)	116.07 ± 51.38	446.08 ± 358.98	134.77 ± 32.32	91.01 ± 23.36
Diclofenac	*t*_1/2_ (h) ^d)^	1.97 ± 0.35	3.04 ± 1.42	3.82 ± 1.65	5.57 ± 3.21
	*T*_max_ (h)	0.60 ± 0.78	0.69 ± 0.88	0.28 ± 0.11	1.33 ± 1.67
	*C*_max_ (μg/mL)	5.98 ± 3.06	0.63 ± 0.28	10.76 ± 2.74	43.69 ± 14.61
	AUC_all_ (μg·h/mL)	7.41 ± 1.35	1.46 ± 1.30	9.44 ± 1.10	125.81 ± 46.49
	AUC_∞_ (μg·h/mL)	7.51 ± 1.38	1.58 ± 1.44	10.1 ± 1.35	129.86 ± 44.66
	AUC_DIC_/AUC_ACE_^a)^	3.04 ± 1.06	2.03 ± 0.24	4.64 ± 1.32	4.33 ± 0.85
Esomeprazole	*t*_1/2_ (h)	1.69 ± 0.75	5.14 ± 5.30	3.87 ± 2.21	3.72 ± 0.38
	*T*_max_ (h)	1.80 ± 3.47	0.21 ± 0.08	0.15 ± 0.14	0.21 ± 0.08
	*C*_max_ (ng/mL)	15.43 ± 3.36	13.54 ± 4.08	57.14 ± 25.94	319 ± 136.81
	*C*_max_ (ng/mL)/D (mg/kg) ^e)^	3.86 ± 0.84	3.39 ± 1.02	14.28 ± 6.49	15.95 ± 6.84
	AUC_all_ (ng·h/mL)	33.83 ± 10.1	27.28 ± 6.43	27.68 ± 13.39	143.85 ± 48.71
	AUC_all_ (ng·h/mL)/D (mg/kg)	8.46 ± 2.53	6.82 ± 1.61	6.92 ± 3.35	7.19 ± 2.44
	AUC_∞_ (ng·h/mL)	42.09 ± 19.27	37.03 ± 11.85	30.91 ± 12.76	151.35 ± 51.65
	AUC_∞_ (ng·h/mL)/D (mg/kg)	10.52 ± 4.82	9.26 ± 2.96	7.73 ± 3.19	7.57 ± 2.58
	CL/F (mL/min/kg)	1786.9 ± 670.64	1910.9 ± 518.68	2442.37 ± 884.54	2356.49 ± 602.56

^a)^ ANOVA (*p* < 0.05, post hoc: Tukey, B vs. C); ^b)^ ANOVA (*p* < 0.05, post hoc: Tukey, C vs. A, B, D); ^c)^ ANOVA (*p* < 0.05, post hoc: Tukey, B vs. A, C, D); ^d)^ ANOVA (*p* < 0.05, post hoc: Tukey, A vs. D; ^e)^ ANOVA (*p* < 0.05, post hoc: Tukey, A, B vs. C, D); * Statistical significance was only presented for dose-independent parameters.

**Table 3:** Non-compartmental pharmacokinetic parameters of aceclofenac, diclofenac, and esomeprazole obtained after oral administration of aceclofenac (20 mg/kg) and esomeprazole (4 mg/kg) to rats under fasted and fed state.

Compound	Parameters	Fasted (*n* = 5)	Fed (*n* = 4)
Aceclofenac	*t*_1/2_ (h) *	2.13 ± 0.30	0.81 ± 0.21
	*T*_max_ (h)	0.57 ± 0.80	0.65 ± 0.91
	*C*_max_ (μg/mL)	2.53 ± 0.57	2.27 ± 1.38
	AUC_all_ (μg·h/mL)	3.35 ± 1.72	2.95 ± 0.41
	AUC_∞_ (μg·h/mL)	3.53 ± 1.89	2.97 ± 0.41
	CL/F (mL/min/kg)	116.07 ± 51.38	114.1 ± 17.25
Diclofenac	*t*_1/2_ (h)	1.97 ± 0.35	2.22 ± 0.59
	*T*_max_ (h)	0.60 ± 0.78	0.69 ± 0.88
	*C*_max_ (μg/mL)	5.98 ± 3.06	3.12 ± 1.91
	AUC_all_ (μg·h/mL)	7.41 ± 1.35	6.36 ± 1.27
	AUC_∞_ (μg·h/mL)	7.51 ± 1.38	6.56 ± 1.31
	AUC_DIC_/AUC_ACE_	3.04 ± 1.06	2.57 ± 0.27
Esomeprazole	*t*_1/2_ (h) *	1.69 ± 0.75	3.18 ± 1.02
	*T*_max_ (h)	1.80 ± 3.47	0.40 ± 0.29
	*C*_max_ (ng/mL) *	15.43 ± 3.36	4.84 ± 0.94
	AUC_all_ (ng·h/mL) *	33.83 ± 10.10	13.96 ± 4.82
	AUC_∞_ (ng·h/mL)	42.09 ± 19.27	17.26 ± 4.42
	CL/F (mL/min/kg)	1786.9 ± 670.64	4061.35 ± 1041.26

* *t*-test (*p* < 0.05, Fasted vs. Fed).
